# Simultaneous Detection of FISH Signals and Bromo-Deoxyuridine Incorporation in Fixed Tissue Cultured Cells

**DOI:** 10.1371/journal.pone.0004483

**Published:** 2009-02-16

**Authors:** Daniela Moralli, Zoia L. Monaco

**Affiliations:** Wellcome Trust Centre for Human Genetics, University of Oxford, Oxford, United Kingdom; Brunel University, United Kingdom

## Abstract

FISH (Fluorescence in situ hybridization) is a powerful technique that detects and localises specific DNA sequences on metaphase chromosomes, interphase nuclei or chromatin fibres. When coupled to BrdU (5-Bromo 2-deoxy-uridine) labeling of newly replicated DNA, the replication properties of different DNA sequences can be analysed. However, the technique for the detection of BrdU incorporation is time consuming, and relies on acidic pH buffer treatments, that prevent use of pH sensitive fluorochromes such as FITC (Fluoro-isothiocianate) during FISH. In this work, we describe a simplified protocol that allows the simultaneous detection of FISH signals and BrdU incorporation. Since the technique does not involve paraformaldehyde for cell fixation, or formamide for denaturation of the target DNA and in post-hybridisation washes, it represents a safer alternative to classical FISH techniques.

## Introduction

The replication of DNA in eukaryotic cells is tightly regulated and time controlled. Euchromatic regions, containing actively expressed genes, are generally replicated in the early stages of the S phase, while non active genes preferentially cluster in areas that are replicated in later stages [1]. Constitutive heterochromatin also replicates towards the end of the DNA synthesis. The variability in replication patterns is seen for example, on the X chromosomes in female mammalian cells. The active copy of the X chromosome is replicated alongside the other autosomes, depending on its gene content, but the inactive copy is the last chromosome to be replicated in the S phase [2]. It has been observed that the difference in replication timing often corresponds to a different spatial localisation of DNA sequence in the nuclear architecture [1]. Moreover, incorrect replication timing of characteristic sequences has been associated with some human diseases such as DiGeorge, Velocardiofacial and Roberts syndromes [3], [4].

The study of DNA replication timing using cytological preparations therefore represents an important tool for the analysis of complex and tightly regulated cellular processes. The analysis includes incorporating BrdU (5-Bromo 2-deoxyuridine), an analogue of thymidine, into actively growing cells and monitoring uptake by DNA sequences using FISH [5]. If BrdU is administered for a short period of time (pulse), it will only be incorporated into the DNA that is replicating during that time. Thus, using specific FISH probes, it is possible to characterise when the specific loci of interest are replicating. The BrdU can be incorporated for longer periods including one or more complete replication cycles, to study processes such as mitotic recombination [6].

Standard replication timing protocols [7]–[9] suggest that following harvesting and fixation in paraformaldehyde of the cells, the BrdU signals can be visualised using specific antibodies. However, due to the position of BrdU residues in the DNA structure, time consuming pre-treatments are necessary to expose them to the antibody. These consist of a lengthy incubation in high concentration HCl (1N and 2N), followed by treatment in borate buffer. The BrdU signal is then detected using a primary antibody, and a fluorochrome conjugated secondary antibody. Following a second incubation in paraformaldehyde to fix the primary and secondary antibody, FISH experiments with specific probes are then conducted to identify the DNA of interest. The cellular DNA is denatured by incubation in 70% formamide, at 70–80 C, before application of the FISH probe. Overall, these techniques are lengthy and time consuming and involve toxic substances such as paraformaldehyde and formamide which require some of the steps to be carried out under chemical hoods. In addition, the HCl treatments may have an effect on some fluorochromes, such as FITC, which is highly sensitive to acidic pH, and if the borate buffer incubation is not conducted appropriately the fluorescence of these molecules is completely lost.

In this work, we describe an alternative technique which allows the simultaneous detection of BrdU incorporation and FISH signals. It is a simpler and less time consuming procedure, and offers a highly reliable and viable alternative technique compared to the standard traditional method described above.

## Methods

### Tissue culture

Human AG6-1 cells (derived from HT1080 cells) [10] and murine LAMF4-9 cells [11] (derived from LA9 cells), both containing a human artificial chromosome (HAC) chromosome, were grown in DMEM medium, 10% FCS. The cells were synchronized by adding aphidicolin at a final concentration of 3 µg/ml for 16 hours. The block was released by washing the cells twice in complete medium, and then BrdU was added to the culture at a final concentration of 40 µM, and left for 15 minutes, for pulse labeling, or for the whole length of the S phase (9 hours).

#### Cell harvesting

At the time of harvesting, the cells were detached by mild trypsinization, swollen in 75 mM KCl hypotonic solution for 6 minutes, and fixed twice in suspension for 15 minutes each in cold methanol∶acetic acid 3∶1. A volume of 50 µl of cell suspension was dropped onto clean slides and allowed to air dry. Chromatin fibres were prepared as described elsewhere [10].

#### Probe labeling

The FISH probes (chromosome 17 alpha satellite (alphoid) DNA, or pBeloBAC 11 vector DNA) were labelled by nick translation of the DNA, incorporating either digoxigenin-11 dUTP (Roche) or biotin-16-dUTP (Roche), using a commercial kit (Nick translation system, Invitrogen). DNA was resuspended at 10 ng/µl in hybridization buffer (50% formamide, 10% dextran sulphate, in 2×SSC pH 7.0).

#### FISH and BrdU detection, standard protocol

The cells on slides were incubated in HCl 1N for 10 minutes on ice, followed by a 30 minute incubation in HCl 2N at 37°C. Next, the slides were placed in Borate buffer 0.1 M for 15 minutes at room temperature. Following a brief wash in PBS, 0.1% Triton×100, the cells were incubated in sheep anti BrdU antibody (Abcam AB1893), for 30 minutes at 37°C. After 3 washes in PBS/Triton ×100, a secondary anti-sheep antibody, either FITC, rhodamine or CY5 conjugated, was applied. Incubation and washes were carried out as above. Next, the cells were fixed in 2% paraformaldehyde in PBS, for 10 minutes, and the DNA was denatured in 70% formamide in 2×SSC for 2 minutes at 80°C. The slides were then washed 1× in 2×SSC buffer at room temperature prior to FISH. In parallel, 10–15 µl of DNA FISH probe was denatured at 85°C for 8 minutes, and the probe was applied to the cells under a coverslip on the glass slide. The FISH was carried out overnight for 16 hr at 37°C. The following day, the slides were washed 3× in 0.1×SSC at 65°C for 5 minutes each. The FISH probe signals were visualised by incubating the cells in rhodamine anti-digoxigenin antibody or FITC conjugated avidin for 30 minutes at 37°C. DNA was counterstained with DAPI, and the slides were mounted in antifading solution. The image analysis was carried out using an Olympus BX60 microscope for epifluorescence equipped with a Sensys CCD camera (Photometrics, USA). Images were collected using either MacProbe 4.3 or Genus Cytovision software.

#### Simultaneous detection of FISH and BrdU signals

In this protocol it is not necessary to detect the BrdU signal prior to FISH. After the harvested cells were dropped on a slide, the DNA on the slide was denatured under a coverslip in buffer containing 10 mM TrisHCl pH 8.0, 50 mM KCl, 5% glycerol at 95°C on a PCR plate for 8 minutes. The slides were then washed 1× in 0.1×SSC buffer for 2 minutes, to remove the coverslip, dehydrated for 2 minutes in 70%, 90%, and 100% ethanol and finally air-dried. The FISH probes were denatured as described above in the standard protocol, and then applied to the cells under a coverslip and left overnight for 16 hours at 37°C. Hybridisation and post-hybridisation washes were carried out as described above. The BrdU and FISH probe signals were visualised simultaneously by incubating the cells in sheep anti BrdU antibody (Abcam AB1893) and rhodamine anti-digoxigenin antibody or FITC conjugated avidin for 30 minutes at 37°C. Following 3 washes in 4×SSC at 42°C, a secondary anti-sheep antibody, either FITC, rhodamine or CY5 conjugated, was applied. The analysis of fluorescent signals and images was done as described.

## Results

In this work, we developed an efficient method to analyse the replication timing of a human artificial chromosome (HAC) containing chromosome 17 alphoid DNA [10]–[11] in human (AG6-1) and murine (LAMF4-5) cells, using bromo-deoxyuridine incorporation and FISH signals in fixed tissue cultured cells. The method allowed consistent and reliable simultaneous detection of both BrdU and FISH signals in S phase cells prepared from each cell line.

Cells were grown under standard conditions, and synchronized by an aphidicolin block to increase the number of cells in S phase. After releasing the block, the BrdU was either added for 15 minutes for pulse labeling, or allowed to remain for the whole length of the S phase (9 hours). At the time of harvesting, the cells were detached, swollen in hypotonic solution, and fixed in cold modified Carnoy's fixative. In this state, cells can be kept at −20°C indefinitely. This allowed the analysis of cells in mitosis and in interphase. To characterise specific DNA sequences at high resolution, chromatin fibres were released from the nuclear structure as described in detail elsewhere [10].

In published standard protocols [7]–[9], following BrdU incorporation and cell fixation, lengthy pretreatments are required to allow visualization of BrdU. Denaturation of the DNA on the slide is carried out using 70% formamide/2×SSC at 80 C prior to hybridisation with a specific DNA probe. In our hands however, we could not reliably detect both BrdU and FISH signals during each experiment following this procedure. In contrast, we were able to detect with high efficiency both BrdU and FISH signals by denaturing the DNA on the slide in a formamide free denaturation buffer. The DNA FISH probes, labeled by nick translation, were then denatured separately and applied to the DNA on the slide. After overnight hybridization, the excess probe and probe bound to non-specific targets was eliminated by stringent washings. The BrdU and FISH probe signals were then visualised simultaneously by incubating with the appropriate antibodies.

**Figure 1:**
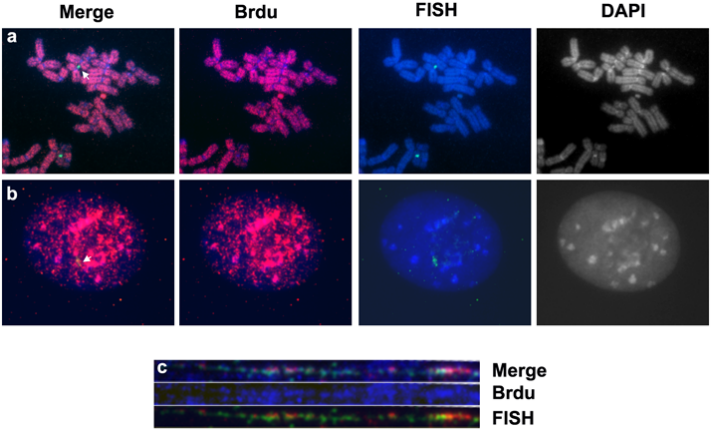
BrdU and FISH signal detection. a) Human AG6-1 chromosome metaphase spread. The probe, chromosome 17 alphoid DNA, identified the HAC (white arrow) and the endogenous chromosome 17. b) Replicating murine LAMF4-5 cell, containing the HAC derived from chromosome 17, probed with chromosome 17 alphoid DNA. In a) and b) the BrdU signal is in red, while the FISH signal is in green. The chromosomes are counterstained in DAPI, blue. The left panels show the merge of all three channels, while in the other panels the channels have been split, to show BrdU and DAPI, FISH and DAPI, and DAPI only, respectively. c) AG6-1 chromatin fibres. BrdU signal is blue, while the red and green signals correspond to different regions of the HAC DNA (chromosome 17 alphoid DNA in green, vector DNA in red). The top panel shows the merge of all three channels. The mid panel shows the BrdU signal only, while the bottom panel shows the FISH signal.

Using our method we obtained consistent and reliable results in 30 different experiments. We observed a strong FISH DNA signal in 97% of the slides, and 90–98% of positive cells exhibited a clear specific FISH DNA signals. The BrdU signal was present in all cells (100%) in each experiment, when BrdU was incorporated during the whole S phase (Table 1, Figure 1). Both the nuclear morphology and chromosome structure was well preserved using these conditions (Figure 1). In comparison, similar experiments done using the standard protocol were not consistent, clear or reliable. In 20 different experiments, only 50% of analysed slides showed FISH signals, and only 70–88% of cells on positive slides contained FISH signals (Table 1). The BrdU signal was detected in 70% of slides, and 95–99% of cells in positive slides contained a BrdU signal (data not shown). Overall, the data indicated that the simultaneous detection of FISH and BrdU incorporation using our method was consistent and highly efficient compared to standard protocols and allowed detection of clear and strong signals.

**Table 1:** Comparison of efficiency of simultaneous detection versus standard technique.

	FISH (%) Efficiency per experiment	BrdU (%) Efficiency per experiment	FISH (%) Efficiency per slide	BrdU (%) Efficiency per slide
Simultaneous detection	97	100	90–98	100
Standard technique	50	70	70–88	95–99

## Discussion

We describe a simple, reliable and efficient cytological technique to study the replication timing of chromosomes in fixed tissue culture cells. We developed a method adapted from standard protocols for detection of replication timing including the incorporation of BrdU and detection of DNA sequences by FISH. Compared to published protocols, there are two major differences in our method. Firstly, we eliminated the extensive pretreatments to expose the BrdU residues, and secondly, the FISH experiments do not require large volumes of formamide in the denaturation buffer. In our study, the denaturation buffer used simultaneously denatured the cell DNA and exposed the BrdU residues, and thus made the HCl and borate buffer treatments redundant. Moreover, the fixation protocol we employed preserved the cells in suspension for future experiments, without the necessity to repeat the cell synchronization and BrdU treatment during each experiment. In repeated independent experiments, we observed a good FISH signal and strong BrdU detection, irrespective of the fluorochromes combinations used to visualize them.

In our hands, the standard protocol based on HCl pretreatments did not prove reliable, and no BrdU signals were observed in about 30% of the experiments undertaken. In some cases, the absence of BrdU signals was attributed to the use of a FITC conjugated secondary antibody. The fluorescence of this fluorochrome is rapidly quenched in acidic conditions. Moreover, in 50% of the experiments, no specific FISH signal was detected. The use of paraformaldehyde, required to preserve the attachment of the anti-BrdU primary and secondary antibody to the target cells, may be responsible for the low efficiency of FISH we observed. The chemical compound forms crosslinks on the DNA structure making the DNA denaturation unreliable and the target DNA unavailable for hybridization with the FISH probe.

In our experience, the protocol developed in this study is highly reliable and allows detection of BrdU and FISH signal on different targets, such as cells in interphase, metaphase chromosomes, and chromatin fibres. It is a simpler, less laborious and time-saving method compared to standard protocols, and represents a significantly improved technique and a step forward in the analysis of DNA replication timing.
